# Solventless Synthesis of Zinc Sulphide Nanoparticles from Zinc Bis(diethyldithiocarbamate) as a Single Source Precursor

**DOI:** 10.1002/open.202400050

**Published:** 2024-05-16

**Authors:** Selina Ama Saah, Patrick Opare Sakyi, Nathaniel Owusu Boadi, Franklyn Addai Tieku, Ampem Kwabena Boampong

**Affiliations:** ^1^ Department of Chemical Sciences University of Energy and Natural Resources Sunyani Ghana; ^2^ Department of Chemistry Kwame Nkrumah University of Science and Technology Kumasi Ghana

**Keywords:** Bandgap, nanotechnology, wurtzite, atom economy, semiconductor

## Abstract

This study explores the synthesis of nanoparticles through the thermal decomposition of single‐source precursors, a method gaining popularity due to its low cost, minimal environmental toxicity, rapidity, scalability, and the ability to form nanoparticles with few defects. Zinc ethyl carbamate was synthesized and characterized using ^1^H NMR and infrared spectroscopy. Its purity was confirmed through microelemental analysis and melting point determination. The melting point of the complex was determined to be 165 °C. The thermogravimetric analyses indicated a one‐step decomposition of zinc ethyl carbamate with a decomposition onset of of 200 °C, yielding a stable ZnS residue. Further thermal decomposition led to the formation of wurtzite phase ZnS nanoparticles, as evidenced by XRD. SEM micrographs displayed mixed spherical, and cubic unevenly sized, polydispersed nanoparticles, while EDX revealed approximately a 1 : 1 Zn to S ratio. Estimated band gap from the Tauc's plot gave 3.93 eV and 3.42 eV for the nanoparticles synthesized at 300 and 400 °C respectively. The wide difference in the band gaps may be as a result of the larger particles observed at 400 °C and the deformations in the sample as observed in the SEM.

## Graphical abstract

In this present study, synthesized zinc ethyl carbamate is thermally decomposed to give wurtzite ZnS nanoparticles. SEM shows polydispersed spherical and cubic structures while EDX reveals approximately a 1 : 1 Zn to S ratio. Tauc's plot give 3.93 eV and 3.42 eV as the band gaps for the nanoparticles synthesized at 300 and 400 °C respectively.

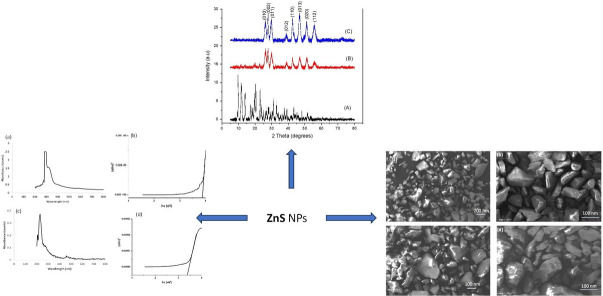

## Introduction

Nanomaterial synthesis has received greater interest in recent years because properties in nanomaterials differ significantly from those in bulk complexes (Boadi et al., 2012; Saah, Boadi, & Wilkins, 2019). Nanotechnology involves more than just reducing dimensions; it allows scientists to explore and harness the distinctive physical, chemical, mechanical, and optical properties exhibited by materials at the nanoscale. In recent years, these materials have emerged as important players in technology, modern medicine and the world (Fujie et al., 2016), (Hussain et al., 2021), (Palanisamy et al., 2020), (Thapa et al., 2019), (Todt et al., 2016), (Veena et al., 2017).

Zinc sulphide (ZnS) has a wide direct band gap of 3.65 eV. It holds great promise as a material suitable for a wide range of optoelectronic devices, including optical coatings, windows for solid‐state solar cells, photoconductors, electrooptic modulators, sensors, field‐effect transistors, transductors, photonic crystal devices and light‐emitting applications that operate within the visible to near‐infrared range (Aguilar et al., 2005; Kumar et al., 2021; Kumar et al., 2023; Liu et al., 2020). ZnS has astounding chemical stability against oxidation and hydrolysis(Sadovnikov, 2019). ZnS nanoparticles are intriguing substances for catalytic applications when they are subjected to harsh environments (Munyai & Hintsho‐Mbita, 2021).

Additionally, ZnS is abundant in nature and non‐toxic.(Maria et al., 2020) Because they eliminate hazardous and organic water contaminants, they can serve as a crucial catalyst in environmental conservation. In wastewater treatment, by photocatalysis, ZnS has been utilized to degrade organic contaminants such as dyes, p‐nitrophenol, and halogenated benzene derivatives (Aziz et al., 2020), (Jothibas et al., 2022).

Zinc sulphide has two phases: zinc blende or sphalerite and wurtzite.(Alafif et al., 2019) Among the two, zinc blende, also referred to as β‐ZnS, is stable at low temperatures, whereas wurtzite, also referred to as α‐ZnS, is stable at temperature>1024 °C (Zhang et al., 2019).

Extensive research has explored the potential use of binary, ternary, and quaternary nanoparticles as absorbers in thin‐film solar cells (Allen & Bawendi, 2008),(Saah et al., 2018). However, binary nanoparticles have been studied more extensively (Ahmadi et al., 2019; Wang et al., 2020). Nanocomposite polymer films that incorporate nanocrystals of inorganic semiconductors hold great potential as materials for the future of photovoltaics, offering advantages such as affordability, lightweight, and flexibility (Wang et al., 2020). One of the promising approaches to the synthesis of nanoparticles is through the use of single‐source approaches. These precursors have several advantages over the dual source approaches. First, including all essential elements within a single molecule or complex simplifies the synthesis process, reducing the required reaction steps and providing a purer substance. This streamlined approach enhances efficiency, saves time, and minimizes the risk of side reactions or impurities (McNaughter et al., 2016), (Saah, Boadi, & Wilkins, 2019). Dithiocarbamate is one of these single‐source precursors. The coordination of the single source precursor, dithiocarbamate, occurs through the sulphur atom and the nitrogen atom of the ligand, forming a chelate complex (Ajibade et al., 2023), (Saah, Boadi, Adu‐Poku, et al., 2019). Zinc dithiocarbamate is prominent in this complexes class and has found relevance in many industrial and biological processes. The methods for obtaining nanoparticles from single‐source precursors include solvent and solventless techniques. The melt method, an example of a solventless technique, involves heating the precursor to a specific temperature, decomposing and releasing zinc and sulphur species (Saah et al., 2018). These species react with each other to form ZnS nanoparticles. This synthesis method simplifies the synthetic process since it does not require complicated treatments and ensures the high purity of the compound obtained, its affordability, and its ability to be applied to various materials (Lewis et al., 2015). The reaction conditions, such as temperature and time, are carefully optimized to obtain nanoparticles with desired size, shape and properties (McNaughter et al., 2016). The melt method, particularly offers several compelling benefits for synthesizing nanoparticles. A standout advantage is the superior purity of the resultant nanoparticles. By sidestepping the solvents commonly used in aqueous processes, the method substantially mitigates the risks of contamination. Moreover, this approach allows for diligent control over the size and morphology of the nanoparticles. By simply adjusting the cooling rate, one can tailor the characteristics of the nanoparticles; for instance, a quicker cooling often yields smaller particles. Notably, these nanoparticles demonstrate commendable resilience under high temperatures, a direct consequence of their high‐temperature synthesis environment (Saah et al., 2018). Furthermore, the melt method ensures an even distribution of elements, resulting in ZnS nanoparticles of consistent composition. Beyond these merits, one of the important attributes of the melt method is its versatility (Saah, Boadi, Adu‐Poku, et al., 2019). In essence, the melt method, when applied with zinc bis(diethyldithiocarbamate), is an innovative, efficient, and adaptable avenue for producing high‐quality ZnS nanoparticles that are well‐suited for sectors where the purity and attributes of nanoparticles are paramount.

## Materials and Methods

Sodium diethyldithiocarbamate (ACS reagent, ≥97.0 %, pellets), and zinc acetate dihydrate (99.999 % trace metals basis) were used as received from Sigma Aldrich.

### Instrumentation

The Thermogravimetric analysis was conducted using the SDT Q600 V20.9 Build 20, employing a 10 °C min^−1^ heating rate from 30–600 °C under nitrogen gas. Micro elemental analysis was performed on the Flash 2000 elemental analyzer, while Thermo iCap 6300 Inductively Coupled Plasma Optical Emission Spectroscopy (ICP‐OES) was used for further elemental analysis. The ^1^H NMR analysis utilized the Ultrashield Advance Bruker 500 MHz NMR. The melting point was determined using the Stuart Scientific melting point apparatus. For crystallography, a Bruker D2 Phaser diffractometer with Cu Kα radiation (λ=1.5418 Å) was employed at 40 kV and 40 mA at room temperature. ZnS nanoparticles were scanned between 10° and 80° with a step size of 0.02° and a dwell time of 3 s. The Zeiss SEM EVO MA 10, equipped with an energy dispersive X‐ray (EDX) spectrometer, was used to examine the nanoparticles′ surface morphology and elemental composition. Prior to SEM and EDX analyses, all samples underwent carbon coating using the Edwards coating system E306 A.

### Synthesis of Zinc Bis(diethyldithiocarbamate) Complex

The synthetic procedure was carried out as reported earlier (Saah, Boadi, Adu‐Poku, et al., 2019). Typically, zinc acetate dihydrate (0.72 mmol, 0.3 g) was added dropwise to a stirring solution of aqueous sodium diethyldithiocarbamate solution ligand (1.43 mmol, 0.49 g). The reaction mixture was stirred for 30 min, and the precipitate was filtered, washed with excess distilled water and dried.

### Melt Reaction Synthesis of ZnS Nanoparticles from Zinc Diethyldithiocarbamate

The melt experiment was carried out as reported in the literature. (McNaughter et al., 2016) The zinc diethyldithiocarbamate precursor (0.3 g) was spread evenly in a ceramic boat. The boat was placed in the centre of a quartz tube and was heated under a nitrogen gas flow of 200 SCCM. The furnace (Carbolite) was heated to the required temperature (200, 300 or 400 °C). The quartz tube was inserted into the heated furnace for 30 minutes. After the heating, the furnace was turned off and the deposit was allowed to cool at 10° min^−1^ under nitrogen gas.

## Results and Discussion

The zinc ethyl carbamate was obtained as a pale white solid with a yield of 95 %. This percentage is consistent with earlier reports on metal carbamates, which are noted to give high yields (Lewis et al., 2015; Saah, Boadi, Adu‐Poku, et al., 2019). Also, the precursor exhibited significant stability, ensuring ease of manipulation during experimental phases. The calculated atom economy was 95 %, confirming the synthetic procedure‘s environmental viability.(Saah, Boadi, & Wilkins, 2019) Melting point determination for the zinc ethylcarbamate precursor began melting at 164 °C and fully melted by 166 °C. The melting point was determined to be 165 °C. The precursor was dissolved in deuterated chloroform (CDCl_3_) to analyze hydrogen environments associated with carbons and other atoms. Due to the symmetrical nature of zinc ethyl carbamate, only half of its structure was considered for evaluation. The ^1^H‐NMR spectrum analysis revealed two primary peaks, a triplet and a quartet (Figure S1). The quartet at 3.96 ppm resulted from coupling effects with adjacent hydrogens of the CH_3_ group, indicating a CH_2_ group near an electronegative nitrogen atom. The nitrogen‘s electron‐withdrawing nature influenced the chemical shift to 3.96 ppm. Conversely, the triplet at 1.37 ppm was influenced by splitting caused by two adjacent hydrogens of a CH_2_ group. Microelemental analysis of the zinc ethylcarbamate (C_10_H_20_N_2_S_4_Zn): calc(found); C: 33.19(33.18), H: 5.57(5.58), N: 7.74(7.72), S: 32.06(32.03), Zn: 18.07(18.12).

**Figure 1:**
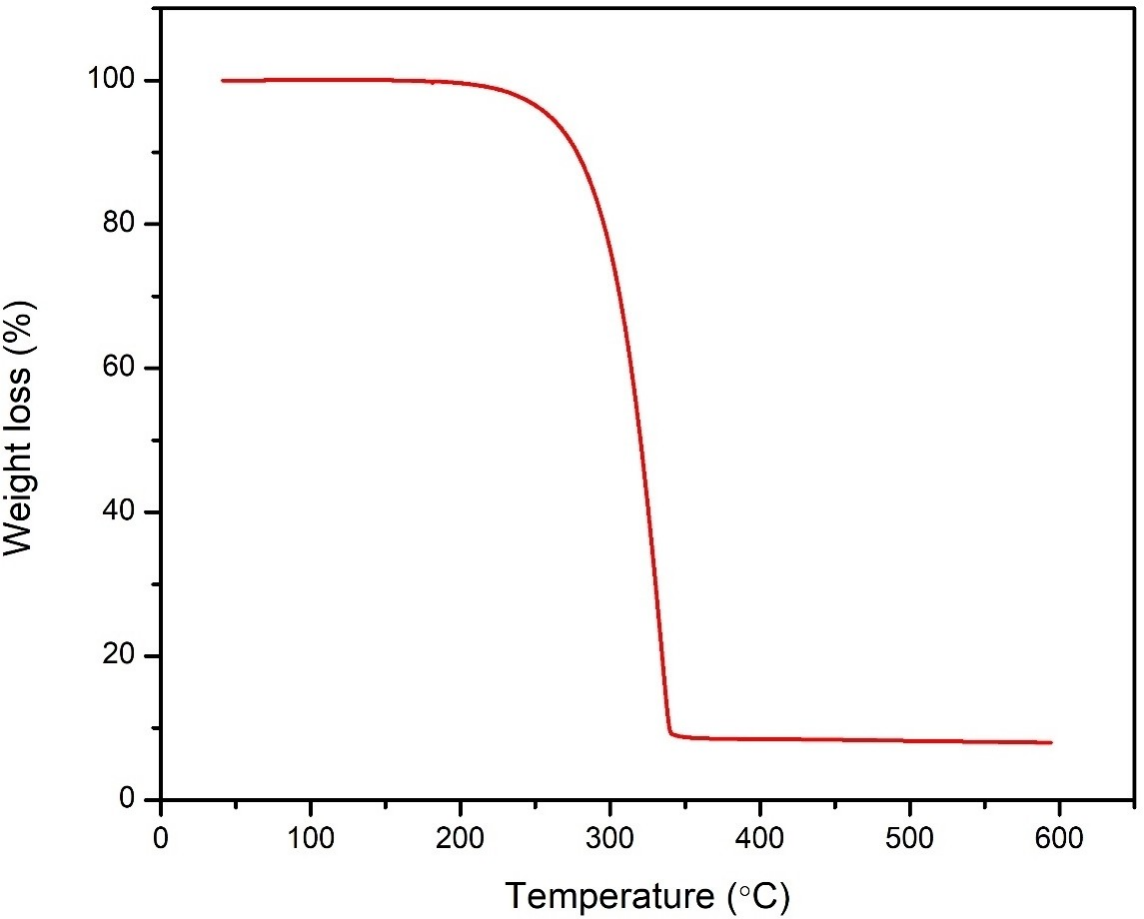
TGA thermograph of zinc ethylcarbamate.

Thermogravimetric analysis was performed to investigate the thermal properties of the zinc ethylcarbamate (Figure 1). The thermogravimetric analysis revealed a one‐step decomposition of zinc ethylcarbamate (Figure 1), aligning with several documented studies. The onset temperature of 200 °C and offset temperature of 330 °C fall within the typical range reported for this compound (190–210 °C and 320–350 °C, respectively.(Shukla & Srivastava, 2017) Similarly, the conclusion that this decomposition corresponds to the loss of both (CH_3_CH_2_NC−) groups agrees with the expected fragmentation pathway involving C−N bond cleavage and ethylamine release.(Onwudiwe & Ajibade, 2012) At the offset temperature, 90 % of the zinc ethylcarbamate had decomposed, corresponding to the two (CH_3_CH_2_NC−) groups attached to the Zn and S atoms.

While our findings broadly agree with existing literature, some noteworthy differences exist. Unlike some studies reporting subtle shoulder peaks or plateaus within the main decomposition range, our thermogram showed a relatively smooth single‐step process (Liu et al., 2019), (Shukla & Srivastava, 2017). This might be attributable to variations in heating rate, sample purity, or instrument sensitivity.

**Figure 2:**
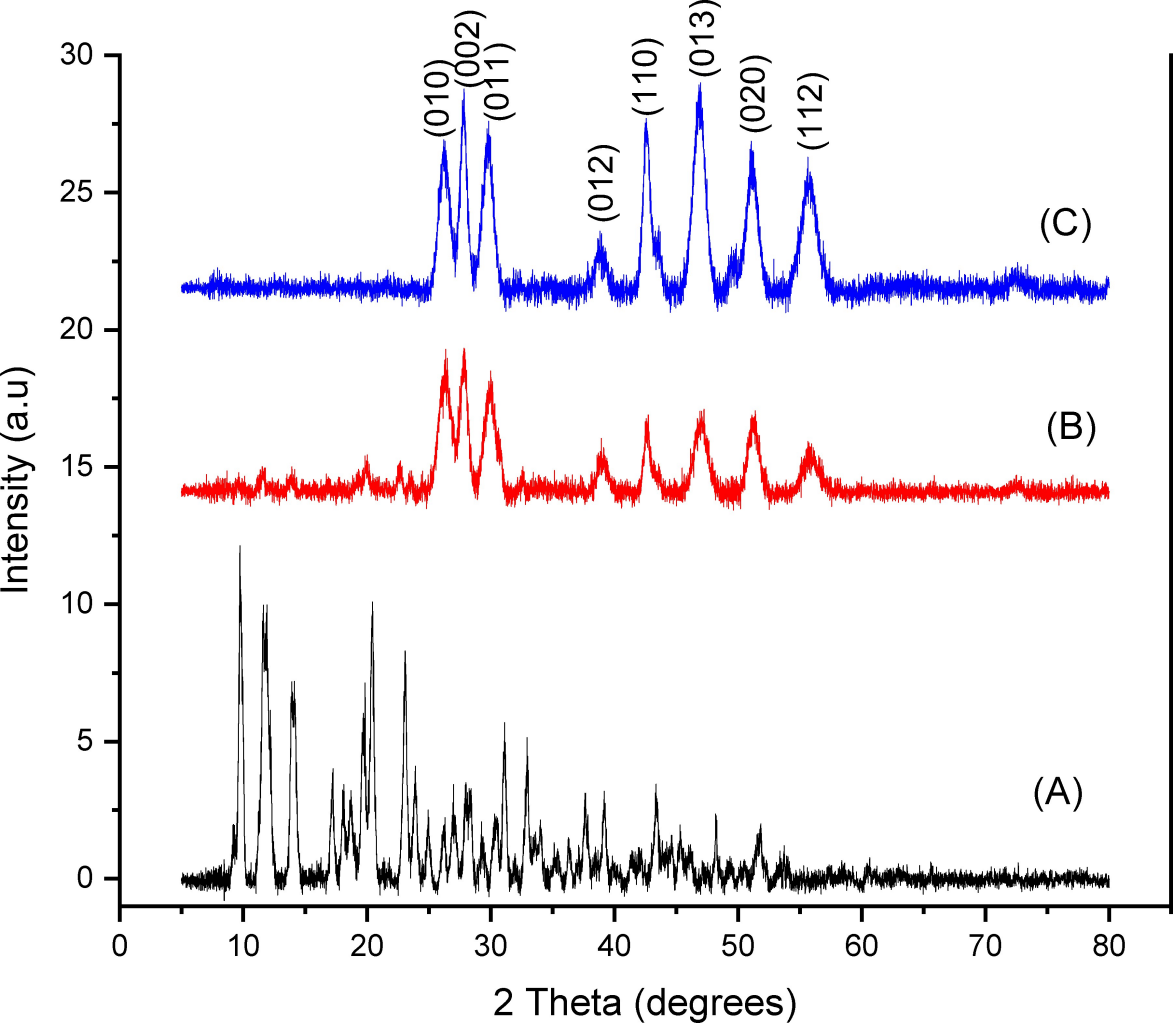
p‐XRD diffraction patterns of ZnS nanoparticles at (A) 200 °C, (B) 300 °C and (C) 400 °C.

The nanoparticles obtained from the melt decomposition of the zinc ethylcarbamate single source precursor at 200, 300 and 400 °C were analyzed by XRD. The XRD pattern at 200 °C exhibits numerous weak peaks alongside some peaks matching ZnS (Figure 2). This observation aligns with the thermogravimetric analysis (TGA) indicating incomplete decomposition of the zinc ethylcarbamate precursor at this temperature. The weak peaks likely correspond to residual precursor or amorphous intermediates formed during early decomposition stages. This finding corroborates the TGA data, where the onset of decomposition was observed at around 200 °C, but complete conversion to ZnS was not achieved until higher temperatures.

In contrast, the XRD patterns at 300 °C and 400 °C demonstrate well‐defined, sharp peaks which can be attributable to the wurtzite ZnS phase. However, at 300 °C, relatively smaller peaks were observed at 2 theta values below 25°. These smaller peaks were fully absent when the temperature was increased to 400 °C. This signifies the complete decomposition of the precursor and formation of crystalline ZnS nanoparticles at this temperature. No evidence of secondary phases or impurities is observed in the patterns, indicating high purity of the synthesized ZnS. This suggests efficient conversion of the precursor and effective removal of decomposition byproducts at higher temperatures.

Scherrer's equation analysis reveals a slight increase in crystallite size from 52.04 nm at 300 °C to 54.01 nm at 400 °C along the (002) plane. While the increase is modest, it suggests that the elevated thermal treatment at 400 °C might have facilitated some crystal growth and Ostwald ripening within the formed ZnS nanoparticles. However, the crystallite size remains relatively small even at 400 °C, hinting at potential advantages in applications requiring high surface area and tunable morphologies (Lewis et al., 2015; McNaughter et al., 2016).

**Figure 3:**
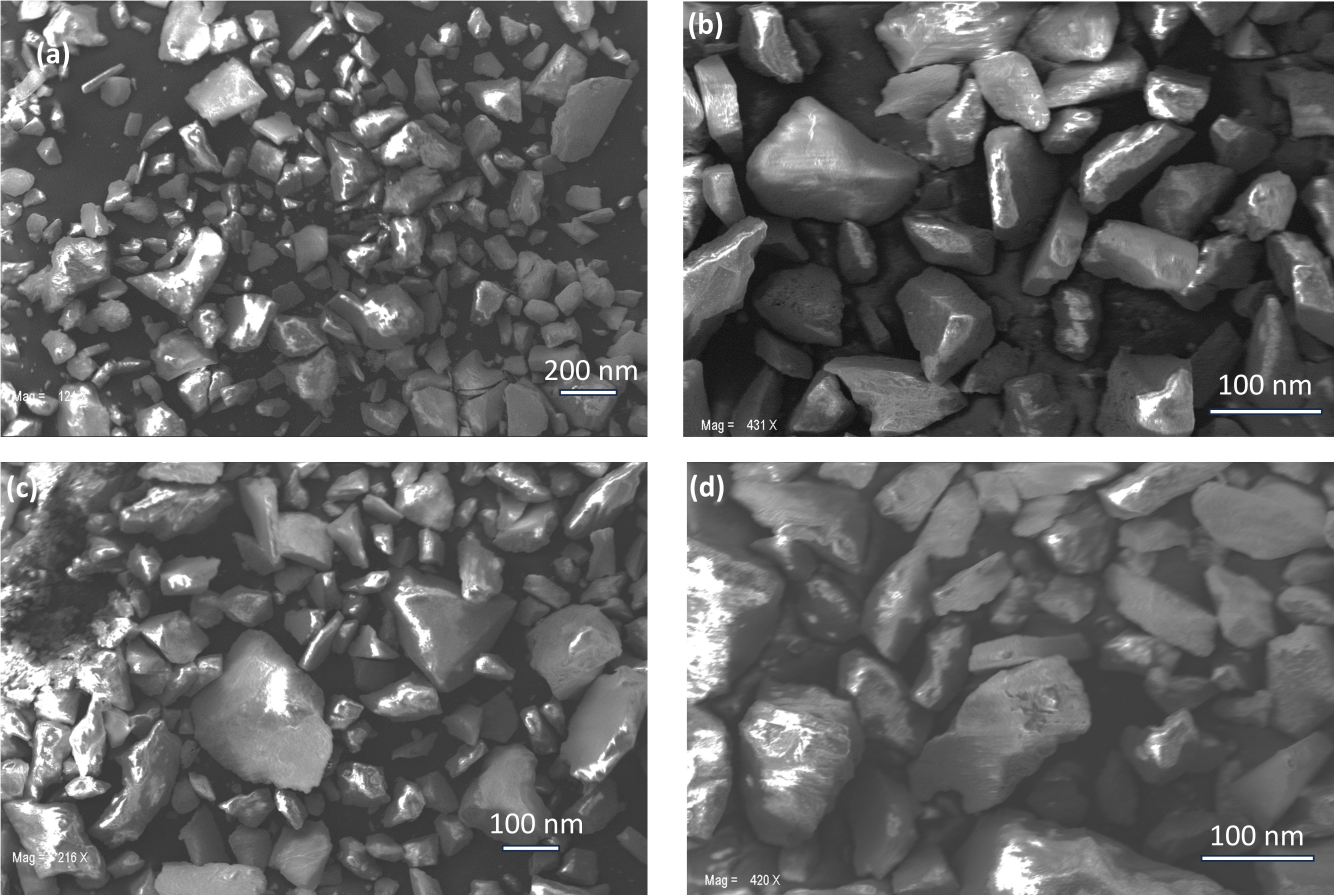
SEM images of ZnS nanoparticles obtained at (a,b) 300 °C and (c,d) 400 °C.

Morphological analyses of the ZnS nanoparticle under a Zeiss SEM EVO MA 10 revealed near‐cubic morphology for the majority of ZnS nanoparticles, consistent with the wurtzite structure observed in XRD. The average size was around 80 nm, but a significant size distribution was present, with some reaching up to 220 nm (Figure 3D). This polydispersity suggests limited growth control, likely due to the absence of dedicated stabilizing agents during synthesis (Saah, Boadi, Adu‐Poku, et al., 2019). While the ethyl groups attached to sulfur might offer some stabilization, their effectiveness seems insufficient and minimal for uniform crystal growth. This variation in crystallite size could potentially impact properties like bandgap, surface area, and catalytic activity, warranting further investigation.

**Figure 4:**
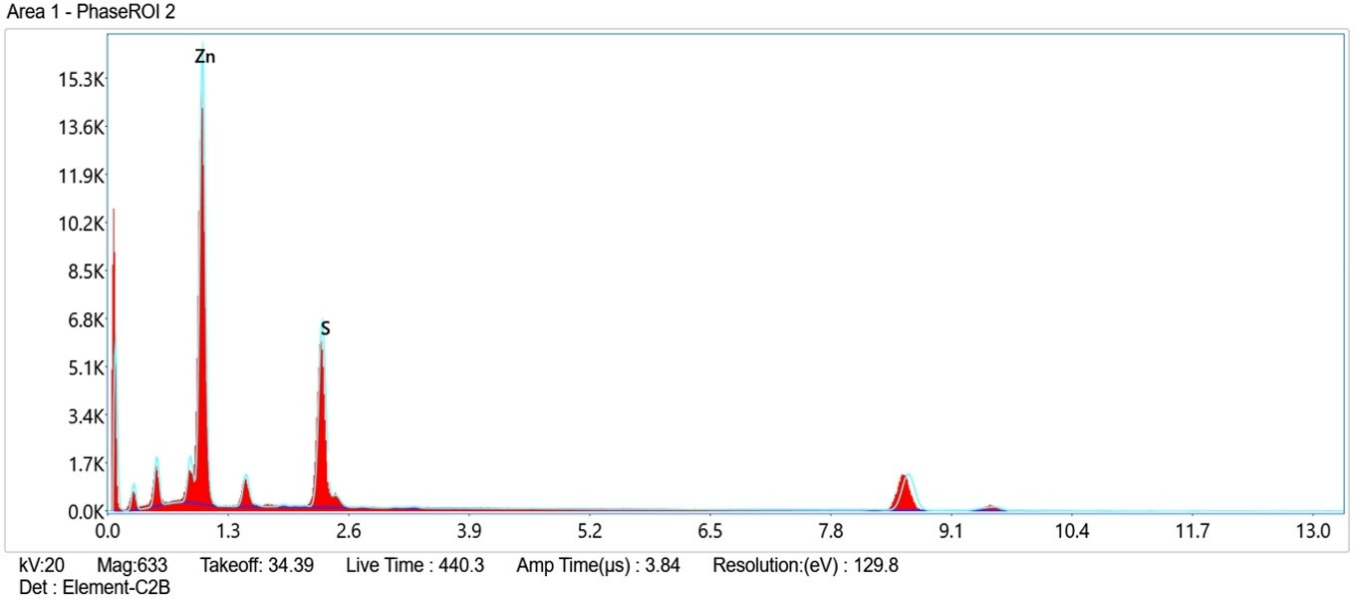
EDX spectrum of ZnS nanoparticles obtained at 300 °C.

EDX analysis (Figure 4) confirmed the elemental composition of the nanoparticles, with characteristic peaks for Zn and S present in a near 1 : 1 stoichiometry (51 % Zn and 49 % S). This further supports the successful conversion of the precursor and reinforces the XRD identification of the wurtzite phase. Deviations from perfect stoichiometry might occur due to trace impurities or surface oxidation, and future work could explore the impact of these on the nanoparticles′ properties and functionality.

**Figure 5:**
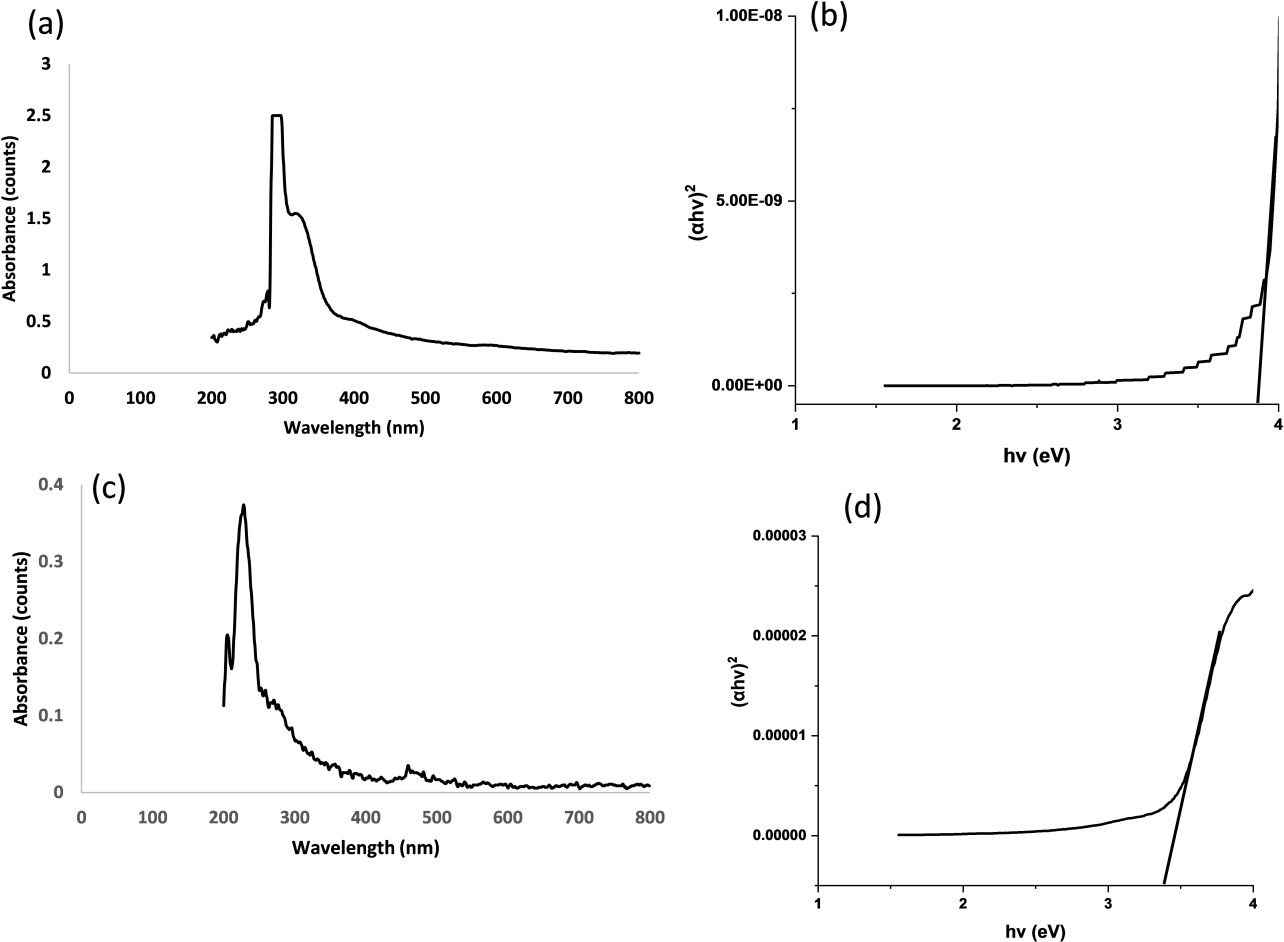
(a) UV vis spectrum and (b) Tauc plot of ZnS nanoparticles obtained at 300 °C, (c) UV vis spectrum and (d) Tauc plot of ZnS nanoparticles obtained at 400 °C.

UV‐Vis spectroscopy was utilized to investigate the optical properties of the wurtzite‐structured ZnS. Using the Tauc plot, the bandgap of wurtzite ZnS at 300 °Cwas determined to be 3.93 eV which blue‐shifted from the 3.65 eV reported the bulk ZnS (Figure 5). Kumar and co‐workers (2021) reported a band gap of 3.74 eV for ZnS nanosphere prepared using a hydrothermal technique. (Kumar et al., 2021). However, at 400 °C the band gap was red‐shifted from 3.65 eV to 3.42 eV (Figure 5) which is similar to the band gap of ZnS thin films obtained from thermal vaporization method (Mezan et al., 2018).

## Conclusions

This study successfully synthesized and characterized a high‐purity, environmentally friendly zinc bis(diethyldithiocarbamate) single‐source precursor. This precursor served as a foundation for efficiently generating wurtzite ZnS nanoparticles through a solventless melt method, as confirmed by X‐ray diffraction analysis. While SEM images revealed a predominantly cubic morphology, the nanoparticles exhibited polydispersity. Energy‐dispersive X‐ray spectroscopy corroborated a near 1 : 1 stoichiometric ratio of Zn to S within the particles, solidifying their structural integrity. The Tauc plots estimated band gaps of 3.93 eV and 3.42 eV for the ZnS nanoparticles synthesized at 300 °C and 400 °C, respectively.

This work highlights the potential of this green precursor for the reliable and efficient synthesis of high‐quality wurtzite ZnS nanomaterials. The unique properties of these nanoparticles, including their band gap and morphology, suggest potential applications in photocatalysis, solar cells, etc. However, future research is warranted to address the observed polydispersity in particle size. Investigating the influence of various reaction parameters, such as temperature and time, on particle size and morphology could be a valuable next step. Additionally, exploring methods to tune the photocatalytic properties of these nanoparticles for specific applications would be an exciting avenue for further exploration. By addressing these limitations and delving deeper into their functionalities, this research paves the way for developing ZnS nanomaterials with tailored properties for future technological advancements.

## Data Availability

Supplementary data will be available from the corresponding author upon request.

## Conflict of interests

The authors declare that they have no conflicts of interest.

1

## Supporting information

As a service to our authors and readers, this journal provides supporting information supplied by the authors. Such materials are peer reviewed and may be re‐organized for online delivery, but are not copy‐edited or typeset. Technical support issues arising from supporting information (other than missing files) should be addressed to the authors.

Supporting Information

## Data Availability

The data that support the findings of this study are available from the corresponding author upon reasonable request.
