# Supplementary material for: Solventless Synthesis of Zinc Sulphide Nanoparticles from Zinc Bis(diethyldithiocarbamate) as a Single Source Precursor
Source: ChemistryOpen. 2024 May 16;13(6):e202400050. doi: 10.1002/open.202400050 (PMC11164025; doi:10.1002/open.202400050)
Supplement: Supplementary file 1 — Supporting Information [file OPEN-13-e202400050-s001.pdf]

ChemistryOpen

Supporting Information

Solventless Synthesis of Zinc Sulphide Nanoparticles from Zinc Bis(diethyldithiocarbamate) as a Single Source Precursor

Selina Ama Saah,* Patrick Opare Sakyi, Nathaniel Owusu Boadi, Franklyn Addai Tieku, and Ampem Kwabena Boampong

Solventless synthesis of zinc sulphide nanoparticles from zinc bis(diethyldithiocarbamate) as a single source precursor

Selina Ama Saah[a*], Patrick Opare Sakyi[a], Nathaniel Owusu Boadi[b], Franklyn Addai Tieku[a], Ampem Kwabena Boampong[a]

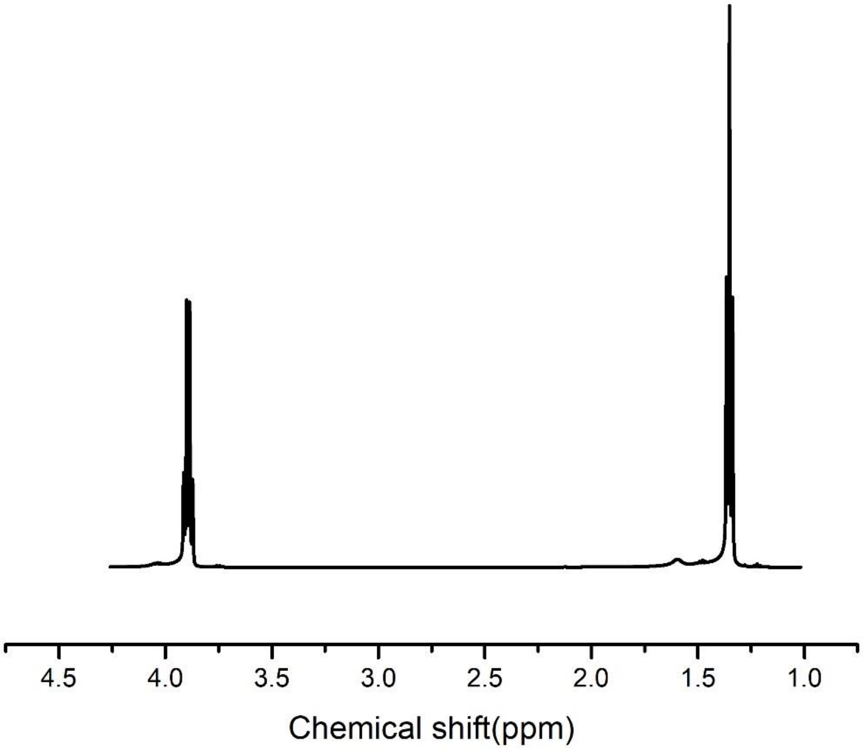

Figure S1: ^{1}H-NMR spectrum of zinc ethylcarbamate
